# Gonadotropin-releasing hormone agonist combined with hormone replacement therapy does not improve the reproductive outcomes of frozen-thawed embryo transfer cycle in elderly patients: a retrospective study

**DOI:** 10.1186/s12958-020-00626-8

**Published:** 2020-07-15

**Authors:** Mei Dong, Li Sun, Li Huang, Yanhong Yi, Xiqian Zhang, Ying Tan, Ge Song, Liling Liu, Fu Wei, Fenghua Liu

**Affiliations:** 1 Department of Reproductive Medical Center, Guangdong Women and Children Hospital, No. 521 Xingnan Road, Guangzhou, 511400 Guangdong Province China; 2 Department of Reproductive Medicine Center, Family Planning Special Hospital of Guangdong Province, Guangzhou, China; 3 Department of Reproductive Medicine and Genetics Center, the People’s Hospital of Guangxi Zhuang Autonomous Region, 6 Taoyuan Road, Nanning, China

**Keywords:** Gonadotropin-releasing hormone agonist, Hormone replacement therapy, Frozen-thawed embryo transfer, Elderly patients

## Abstract

**Background:**

With the rapid development of whole embryo freezing technology, more and more frozen-thawed embryo transfer (FET) was used in assisted reproductive technology. However, the best FET program for elderly women has not been finalized. We intended to explore the reproductive outcomes of traditional hormone replacement treatment and a gonadotropin-releasing hormone agonist (GnRHa) combined with hormone replacement treatment in the frozen-thawed embryo transfer cycle of elderly patients.

**Methods:**

In this retrospective analysis, we analyzed 1264 elderly patients (aged 38 years or older) who underwent FET at three reproductive centers between 2015 and 2017. According to the endometrial preparation protocol, we divided the patients into a GnRHa combined with hormone replacement treatment (GnRHa-HRT) group and traditional hormone replacement treatment (HRT) group. The clinical pregnancy, ongoing pregnancy, live birth, and abortion rates were compared between groups.

**Results:**

One-way analysis of variance of the two groups revealed no significant difference in the clinical (33.58% vs. 37.15%) and ongoing pregnancy rates (19.40% vs. 25.10%) between the GnRHa-HRT and HRT groups. The live birth rate (17.54% vs. 24.10% *p* = 0.0229) of the GnRHa-HRT group was lower than that of the HRT group, whereas the abortion rate (45.56% vs. 32.97% *p* = 0.0252) was higher than that of the HRT group. However, multivariate analysis showed no significant difference in the live birth rate (*p* = 0.1333) or abortion rate (*p* = 0.1881) between the GnRHa-HRT and HRT groups. The number of embryos transferred, level of the embryo, and age and ovarian reserve of the patient significantly affected final reproductive outcomes.

**Conclusion:**

A GnRH agonist combined with hormone replacement therapy did not improve the reproductive outcomes of frozen-thawed embryo cycles in elderly patients.

## Background

Since Trouson performed the world’s first frozen-thawed embryo transfer (FET) in 1983 resulting in a successful clinical pregnancy, FET has played an important role in assisted reproduction technology [1]. FET can increase the cumulative pregnancy rate in the single egg retrieval cycle, reduce the occurrence of moderate and severe ovarian hyperstimulation syndrome, and reduce the risk of multiple pregnancy. This method is also simpler and easier to implement than fresh cycles, causing less pain to patients and reducing time and expenses. In countries that strictly implement the single embryo transfer strategy, FET is performed in 50–80% of cases [2]. Compared to the diversity of superovulation promotion schemes, the FET scheme is relatively simple and there is no standard conclusion on the choice of FET schemes. The most commonly used protocol is traditional hormone replacement treatment (HRT) [3, 4].

Administration of a gonadotropin-releasing hormone (GnRH) agonist combined with hormone replacement treatment (GnRHa-HRT), as another FET protocol [5–7], has been shown to be successful in patients with endometriosis and repeated implantation failure and has achieved good reproductive outcomes [8–10]. GnRHa-HRT refers to the application of a GnRH agonist (GnRHa) in the preparation of endometrium to inhibit the surge of luteinizing hormone (LH) before estrogen administration [11, 12]. Since the beginning of endometrial hyperplasia, continued application of estrogen alone has been shown to be sufficient to suppress ovulation through the negative feedback mechanism of the hypothalamic-pituitary-ovarian axis [13]. In the initial stage of estrogen administration alone, the endometrium thickens and is maintained, whereas follicular development is inhibited. Daily progesterone administration is started 5 days before the planned embryo transfer. Estrogen maintains the proliferative phase to keep the endometrium in a pre-ovulatory state until the start of progesterone to induce the endometrium to transform into an embryo-accepting state.

Studies have shown that the pregnancy rate of FET decreases with increasing patient age, particularly in those older than 40 years of age [14]. An increased patient age mainly affects ovarian function [14]. Both the number and quality of eggs obtained from elderly patients are lower than those from younger patients [14, 15]. Some studies have shown that chromosomal abnormalities in embryos in elderly women are significantly increased, leading to reduced pregnancy rates and increased abortion rates [15]. Two systematic reviews and meta-analysis [4, 16] concluded that there is insufficient evidence for recommending specific protocols for endometrial preparation in FET cycles, and few studies have evaluated the choice of endometrial preparation protocols in elderly patients.

This study was performed to compare the effect of HRT protocols and GnRHa-HRT protocols in FET on elderly patients.

## Methods

### Study design and participants

The study included elderly patients (aged 38 years or older) undergoing FET cycle therapy at three centers between January 2015 and December 2017. This retrospective cohort study only included patients undergoing the first embryo transfer after autologous in vitro fertilization and intracytoplasmic sperm injection (*n* = 1264). The following patients were excluded from the study: 1. patients who underwent embryo transfer after preimplantation genetic testing (PGT), 2. patients who used blastocysts derived from previous stimulation cycles (i.e., cryopreserved oocytes and/or donor oocytes), 3. patients whose endometrial thickness did not reach 7 mm on the day of transplantation, 4. natural cycle or ovulation-promoting cycle FET, 5. women aged 45 years or older, and 6. patients who attempted fresh cycle transplantation, had a repeated abortion history, or congenital uterine malformations. The research protocol was approved by the hospital institutional ethics committee (202001043).

### Endometrial preparation before embryo transfer

After completing standard in vitro fertilization and intracytoplasmic sperm injection along with whole embryo freezing, the patient returned after her second menstrual period. On day 3 of spontaneous menses, the patients underwent a baseline transvaginal ultrasound and assessment of serum estrogen, progesterone, and luteinizing hormone to confirm that they were in the early proliferative phase of their menstrual cycle.

In the HRT strategy, patients then began administration of oral estrogen, 2 mg twice daily for 1 week, followed by 3 mg twice daily. Oral estrogen was administered to induce endometrial proliferation while suppressing dominant follicle development. We performed transvaginal ultrasound every week to assess the recipients’ endometrium, with the first ultrasound occurring within 7 days of initiating estrogen supplementation. Serum progesterone was measured at each visit to rule out premature ovulation before initiating progesterone supplementation.

In the GnRHa-HRT strategy, on days 2–3 of the menstrual period, which was the early follicular period, the patient was administered the full 3.75 mg dose of GnRHa We asked the patient to return to the hospital after 28 days, regardless of whether menstrual cramps had occurred during this period. Next, we assess whether the patient had reached pituitary down-regulation status based on ultrasound and hormone levels. The standard criteria use to determine the down-regulation status were estrogen (E2) < 183.5 pmol/L, follicle-stimulating hormone < 5 U/L, luteinizing hormone (LH) < 5 U/L, uterine endometrial thickness < 5 mm, and no large follicles or cysts. After reaching the down-regulation standard, the patient administration of the drug as described for the HRT scheme.

Once the timing of the FET was determined, progesterone in the form of intramuscular or vaginal combined with oral administration of progesterone was performed daily. The route of progesterone supplementation was based on the patient’s preference, as there is no medical indication for the use of one regimen over the other. Patients were administered intramuscular progesterone in oil or vaginally and a combination of oral progesterone, starting at 4 days before FET when transplanting the cleavage embryos, as well as 5 days before FET when transplanting the blastocysts.

After FET, daily estrogen and progesterone administration was continued until a negative pregnancy test was obtained. If pregnancy was achieved, hormone administration was continued until the expected luteoplacental shift in estrogen and progesterone production at approximately 8–9 weeks of gestation.

### Embryo vitrification, thawing, and transfer

Briefly, embryo vitrification was carried out using a Cyrotop carrier system with a solution of dimethyl sulfoxide, ethylene glycol, and sucrose used as a cryoprotectant. For thawing, embryos were transferred into dilution solution in a sequential manner (1–0.5–0 mol/L sucrose).

Cleavage-stage embryos (day 3) were graded according to the Cummins criteria. Grade I and II embryos were classified as high-quality and selected for vitrification. Suboptimal cleavage-stage embryos were placed in extended culture to the blastocyst stage. Quality assessment of blastocyst stage embryos (days 5 and 6) was based on the scoring system of Gardner and Schoolcraft, with embryos graded as R3BB considered as good blastocysts. In all FET cycles, no more than three embryos were transferred. All embryos were thawed on the day of transfer, and post-thaw embryos with R50% blastomeres intact were considered as having survived.

### Outcome parameters

In evaluating which endometrial preparation methods impacted reproductive outcomes, we analyzed the clinical pregnancy, ongoing pregnancy, abortion, and live birth rates. The clinical pregnancy rate per woman was defined as the presence of at least one gestational sac in the uterine cavity on ultrasound at 5 weeks after ET. The ongoing pregnancy rate per woman was defined as evidence of a gestational sac with fetal heart motion at 12 weeks as confirmed by ultrasound. The abortion rate was defined as a loss of clinical pregnancy before the 28th gestational week. The live birth rate per woman was defined as delivery of a live fetus after 24 completed weeks of gestation.

### Statistical analysis methods

**Fig. 1:**
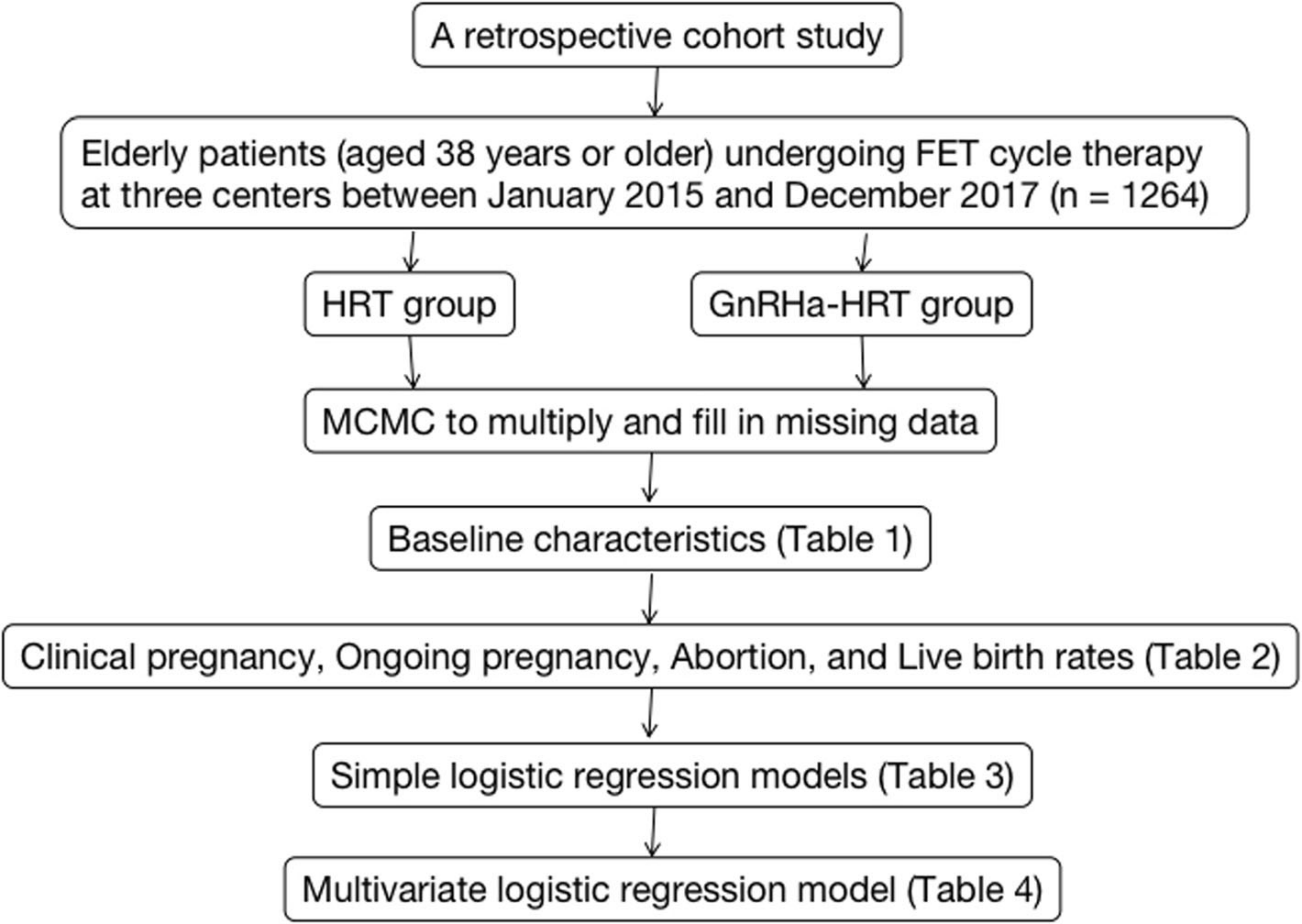
Data collection and analysis method

Our data collection and analysis method is shown in Fig. 1. SAS9.4 software was used for statistical analysis (SAS, Inc., Cary, NC, USA).

We first used the Markov chain Monte Carlo method (MCMC) to multiply and fill in missing data.

Next, we examined the demographic and baseline characteristics of the patients. When continuous variables followed a normal distribution, the mean ± standard deviation was used for statistical description and *t* test was used to compare groups. For non-normally distributed data, the median ± quartile was determined. For statistical description, the nonparametric rank sum test was used to compare groups; for categorical variables, the frequency (percentage) was used for statistical description, and use χ^2^ test to compare groups.

The FET strategy with each potential confounding factor was analyzed by fitting to simple logistic regression models.

Variables showing a *p* < 0.1 in univariate logistic regression were included in the multivariate logistic regression model to identify the impact of different FET treatment regimens on clinical outcomes.

## Results

This was a retrospective study of 6397 patients. Of these, 8 cases were eventually cancelled, 2 cases were lost to follow-up, 3 cases had no transplantable embryos, 3 cases were non-first transplantation cycles, and 6338 cases were completed. Fifty-one patients were over 45 years old, and 6338 patients were finally eligible. Among them, 1264 patients were elderly (38 years or older), 996 underwent HRT-FETs, and 268 underwent GnRHa-HRT-FETs (Fig. 1).

### Baseline characteristics

The baseline characteristics of the patients are detailed in Table 1. There were no significant differences in age, basal follicle-stimulating hormone level, body mass index, number of transferred embryos, blastocyst stage embryos or blastocysts, and fertilization methods between the two groups. Because HRT is more commonly used in clinical applications, the number of patients in the HRT group was significantly greater than that in the GnRHa-HRT group. However, the GnRHa-HRT group had a relatively long infertility period compared to the HRT group (*p* = 0.021). The basic LH level of the GnRHa-HRT group was slightly lower than that of the HRT group (*p* = 0.002), and the intima thickness of its corpus luteum-supported conversion day was slightly higher than that of the HRT group (*p* < 0.001).

**Table 1:** Characteristics of FET cycle in the two study groups

Characteristic		GnRHa-HRT(*n* = 268)	HRT(*n* = 996)	*P* value
Age (y)		40.67 ± 2.07	40.64 ± 2.04	0.804
Years of infertility		6.28 ± 5.27	5.51 ± 4.75	0.021^✱^
BMI (kg/m2)		22.51 ± 2.99	22.72 ± 2.94	0.305
FSH (U/L)		7.24 ± 3.10	7.50 ± 3.47	0.315
LH (U/L)		4.00 ± 2.32	4.36 ± 2.73	0.002^✱^
Intimal thickness		9.85 ± 2.23	9.14 ± 1.93	< 0.001^✱^
No of embryos transferred	1	59(22.01)	258(25.90)	0.146
	2	198(73.88)	677(67.97)	
	3	11(4.10)	61(6.12)	
Embryos transferred	Cleavage stage embryo	152(56.72)	627(62.95)	0.062
	Blastocyst	116(43.28)	369(37.05)	
Fertilization method	IVF	77(28.73)	241(24.20)	0.053
	ICSI	185(69.03)	746(74.90)	
	IVF + ICSI	6(2.24)	9(0.90)	

Values are means±SD or number (percentage) of patients. ^✱^*P* < 0.05 for *t* test.

Abbreviations: *BMI* body mass index; *FSH* follicle-stimulating hormone; *LH* luteinizing hormone; *ICSI* intracytoplasmic sperm injection; *IVF* in vitro fertilization

### Reproductive outcomes

First, we preliminary calculated the fertility outcomes of the two treatment groups and found no significant difference in the clinical pregnancy rate (33.58% vs. 37.15%) or sustained pregnancy rate (19.40% vs. 25.10%) between the GnRHa-HRT and HRT groups. The live birth rate (17.54% vs. 24.10% *p* = 0.0229) of the GnRHa-HRT group was lower than that of the HRT group, whereas the abortion rate (45.56% vs. 32.97% *p* = 0.0252) was higher than that of the HRT group (Table 2).

**Table 2:** Reproductive outcomes of two study groups

Reproductive outcome	GnRHa-HRT	HRT	P value
Clinical pregnancy rate	33.58(90/268)	37.15(370/996)	0.2814
Ongoing pregnancy rate	19.40(52/268)	25.10(250/996)	0.0522
Abortion rate	45.56(41/90)	32.97(122/370)	0.0252*
Live birth rate	17.54(47/268)	24.10(240/996)	0.0229*

Values are percentage (no./no.) of patients. *P < 0.05 for *t* test

### Single-factor analysis of factors related to FET outcomes

Next, to explore the factors influencing FET in elderly patients to facilitate multivariate analysis of variance in adjusting for confounding factors, we performed single-factor analysis of variance of whether the patient was clinically pregnant, whether she was pregnant, whether she had a newborn, and whether she had an abortion. The analysis items included the woman’s age, follicle-stimulating hormone level, antral follicle count (AFC), corpus luteum endometrial thickness, number of transferred embryos, transplant records, and FET scheme. To simplify the table, the results for factors that with no influence on reproductive outcomes are not listed in Table 3. These factors included body mass index, age of infertility, type of infertility, LH level, support day, center name, and fertilization method.

**Table 3:** Single-factor analysis of factors related to FET outcomes

Clinical indicators		Clinical pregnancy rate		Sustained pregnancy rate	
Value		OR value(95% CI)	P value	OR value(95% CI)	P value
Age (y)		0.8(0.753–0.849)	< 0.001***	0.725(0.673–0.781)	< 0.001***
FSH		0.96(0.933–0.988)	0.005**	0.949(0.915–0.984)	0.005**
AFC		1.050(1.029–1.071)	< 0.001***	1.065(1.042–1.089)	< 0.001***
Intimal thickness		1.030(0.974–1.090)	0.301	1.055(0.991–1.124)	0.093
BMI		1.040(1.000–1.081)	0.049	1.036(0.991–1.082)	0.117
No of embryos transferred	1	Ref.		Ref.	
	2	1.494(1.132–1.972)	0.005**	1.238(0.908–1.688)	0.177
	3	1.927(1.141–3.254)	0.014*	1.153(0.629–2.116)	0.645
Embryos transferred	Cleavage stage	Ref.		Ref.	
	Blastocyst	1.786(1.413–2.258)	< 0.001***	1.69(1.301–2.195)	< 0.001***
FET strategy	GnRHa-HRT	Ref.		Ref.	
	HRT	1.169(0.880–1.553)	0.282	1.392(0.996–1.946)	0.053
Clinical indicators		Live birth rate		Abortion rate	
Value		OR value(95% CI)	P value	OR value(95% CI)	P value
Age (y)		0.721(0.668–0.778)	< 0.001***	1.31(1.179–1.454)	< 0.001***
FSH		0.948(0.913–0.984)	0.005**	1.032(0.987–1.080)	0.166
AFC		1.065(1.042–1.089)	< 0.001***	0.953(0.921–0.986)	0.006**
Intimal thickness		1.048(0.983–1.118)	0.149	0.979(0.891–1.076)	0.748
BMI		1.030(0.985–1.076)	0.196	0.989(0.924–1.058)	0.658
No of embryos transferred	1	Ref.		Ref.	
	2	1.194(0.872–1.635)	0.269	1.382(0.838–2.279)	0.205
	3	1.108(0.597–2.057)	0.746	2.157(0.944–4.927)	0.068
Embryos transferred	Cleavage stage	Ref.		Ref.	
	Blastocyst	1.621(1.242–2.115)	< 0.001***	1.043(0.711–1.529)	0.829
FET strategy	GnRHa-HRT	Ref.		Ref.	
	HRT	1.492(1.055–2.111)	0.024*	0.588(0.368–0.939)	0.026*

*P < 0.05, **P < 0.01, ***P < 0.001 for Chi-square test

Abbreviations: *OR* odds ratio; *CI* 95% confidence interval; *FSH* follicle-stimulating hormone; *AFC* antral follicle count; *BMI* body mass index; *FET* frozen-thawed embryo transfer

For the clinical data, age, follicle-stimulating hormone levels, AFC, and BMI significantly affected clinical outcomes (Table 3). In contrast, endometrial thickness on the luteal support day did not impact the clinical outcome of frozen embryo transplantation.

Regarding laboratory data, the number of embryos transferred impacted the clinical pregnancy rate but not the ongoing pregnancy rate, abortion rate, or live birth rate (Table 3). Transplanted blastocysts showed a better clinical pregnancy rate, ongoing pregnancy rate, and live birth rate than cleavage stage embryos, but did not show a worse abortion rate (Table 3).

Regarding the transplantation scheme, the results were the same as those obtained by the *t* test, with no significant difference in the clinical pregnancy rate or ongoing pregnancy rate between the GnRHa-HRT and HRT groups. The live birth rate of the GnRHa-HRT group was lower than that of the HRT group, whereas the abortion rate was higher than that of the HRT group. The detailed statistical information for these results is shown in Table 3.

### Multifactor analysis of factors related to FET outcomes

Logistic regression analysis was performed using live birth rate and abortion rate as dependent variables and age, AFC level, number of transferred embryos, transplantation records, and FET protocol as independent variables.

The results showed that age and the number of embryos transferred significantly affected live and abortion rates. The patient’s AFC level appeared to only affect the live birth rate but not the abortion rate. Compared with cleavage stage embryo transplantation, blastocyst transplantation can increase the live birth rate but does not affect the abortion rate. Between these transplantation strategies, the two endometrial preparation schemes had no effect on the live and abortion rates (Table 4).

**Table 4:** Multifactor analysis results of FET outcome-related factors

Reproductive outcome		Live birth rate		Abortion rate	
Value		OR value(95% CI)	*P* value	OR value(95% CI)	*P* value
Age (y)		0.728(0.672–0.789)	< 0.001***	1.316(1.177–1.471)	< 0.001***
AFC		1.045(1.019–1.071)	0.0006***	0.968(0.934–1.003)	0.0758
No of embryos transferred	1	Ref.		Ref.	
	2	1.937(1.302–2.881)	0.0011**	0.525(0.297–0.928)	0.0267*
	3	2.613(1.712–3.987)	< 0.001***	0.403(0.220–0.739)	0.0033**
Embryos transferred	Cleavage stage embryo	Ref.		Ref.	
	Blastocyst	1.288(0.958–1.731)	0.0936	0.44(0.267–0.726)	0.0013**
FET strategy	GnRHa-HRT	Ref.		Ref.	
	HRT	1.358(0.911–2.024)	0.1333	0.711(0.428–1.181)	0.1881

*P < 0.05, **P < 0.01, ***P < 0.001 for logistic regression

Abbreviations: *OR* odds ratio; *CI* 95% confidence interval; *AFC* antral follicle count; *FET* frozen-thawed embryo transfer

## Discussion

In this study, we found that GnRH agonist combined with HRT did not improve the clinical outcomes of frozen embryo cycles in patients with an advanced age. In contrast, in older patients, factors such as the number of embryos transferred and stage and grade of the transferred embryos determined the outcome of pregnancy.

The effect of age in the fresh cycle on pregnancy outcomes is well-known, whereas domestic and foreign studies of the effect on the pregnancy rate of FETs have shown consistent results [17, 18]. A 2011 study [2] suggested that age is not correlated with the clinical pregnancy rate of FETs as long as there is a sufficient number of high-quality eggs and embryos. However, the number of embryos obtained from women in the fresh cycle is typically small and the quality is obviously reduced. In 2016, a study [14] showed that the clinical pregnancy rate of FETs in the ≥40-year-old group was significantly reduced. Thus, the age of patients was shown to affect ovarian function, not only for the number of eggs obtained, but also the quality of the eggs, and the chromosome numbers in embryos from older women are significantly increased. These factors lead to reduced pregnancy rates.

In the field of reproduction, the main factors affecting FET outcomes are the quality and number of embryos, as well as the thickness and type of endometrium and synchronization of the endometrium [17, 19]. The most controllable factor in patients planning to undergo FET is the patient’s endometrial condition, and improving the endometrial receptivity is a research hotspot. The current commonly used freeze-thaw cycles for preparing the endometrium are natural cycle, hormone replacement cycle, ovulation promotion cycle, and downregulation of the hormone replacement cycle. The downregulation hormone replacement cycle scheme studied in this article involved using a long-acting GnRHa for pituitary down-regulation and using a hormone replacement cycle to prepare the endometrium. This approach avoids abnormal spontaneous ovulation during the hormone replacement cycle with a low transplantation cycle. The endometrium is susceptible to regulation by exogenous hormones and can improve endometrial receptivity. Clinically, downregulation combined with the hormone replacement cycle scheme was gradually applied in the FET cycle. Studies of the planting window and superiority of this scheme in FET have shown controversial results. Our study confirmed that downregulating FET schemes are not advantageous in elderly patients.

Our experimental study design had the following advantages. 1. This is the first large data multicenter retrospective analysis of FET strategies in older patients, and the in vitro fertilization strategy and laboratory conditions did not changed significantly during entire follow-up period. 2. We included patients undergoing FET for the first time, which avoided factors related to repeated implantation failure and bias caused by embryo selection. This may minimize the inclusion of patients with potential problems due to recurrent FET. Moreover, we also avoided biases associated with optimal embryo transfer. Finally, strict exclusion criteria were used to ensure that the duration of estrogen exposure before FET was independent of the patient or cycle characteristics and depended only on patient or physician availability or preferences. 3. The threshold for endometrial thickness on our transformation day was set to 8 mm. Some studies have suggested that an endometrial thickness of ≥8 mm leads to better FET outcomes [5]. 4. In the luteal support dosing regimen, we did not stipulate a uniform and strict dosing route, which was based mostly on the patient’s willingness to administer medication. Numerous studies have suggested that the outcome of FET pregnancy is comparable for any one administration method or combination [20, 21]. 5. In the screening of retrospective cases, we strictly controlled the oocyte extraction and frozen embryo transfer to only 2 menstrual cycles. A previous study [22] showed that the live birth rate was 57.8% when FET was performed after 2–3 months of menstruation, compared to the live birth rate obtained after less than 2 or more than 3 menstrual cycles. This suggests that extending the FET time does not improve the live birth rates.

The results of this study demonstrate the potential advantages of a downregulation scheme when performing the first analysis, although negative results were obtained after performing adjustment analysis. Because previous studies suggested that downregulation schemes are useful in patients with repeated implantation failures, endometriosis, and polycystic ovary syndrome, we performed FET scheme selection of elderly patients and obtained unexpected results. We analyzed whether adjustments were needed in the downregulation scheme. First, we considered whether it the time of estrogen administration after downregulation was sufficient. The literature [23] suggests that prolonging the estradiol action time in the conventional HRT regimen can significantly improve pregnancy outcomes. However, in the GnRHa-HRT regimen, prolonging the estradiol action time did not significantly improve pregnancy outcomes [23]. During periods without pituitary aura-inhibition, clinical pregnancy rates were reduced in patients in the longer estrogen-administered group. However, following prior downregulation with GnRHa, there was no significant difference in the timing of estrogen administration. Studies have shown that prior to beginning progesterone supplementation, previous downregulations prevented the harmful effects of long-term estrogen use [23]. Second, we evaluated whether the cut-off value of endometrial thickness on the conversion day after the downregulation scheme should be adjusted. Prospective studies of a large number of patients are needed to resolve this issue.

There were some limitations to the study. 1. This was a retrospective study and complete randomized double-blind comparison was not possible. 2. Patients were only followed up for 12 weeks in our center. Thus, we were unable to consider the impact of high-risk obstetric factors such as preterm birth on pregnancy outcomes or other exposure factors that caused treatment failure such as tobacco. 3. Experimental research [24, 25] has confirmed that the estradiol level for the previous physiological dose in the previous cycle affects the final FET outcome. 4. In addition, the number of patients included in the GnRHa-HRT group was significantly smaller than the HRT group, which is related to the personal preference of doctors when choosing treatment, and thus this bias is unavoidable.

Further well-designed prospective clinical trials of more analogies of programs, such as natural cycle programs and cycle promotion programs are needed. Additionally, stricter obstetric follow-up should be performed. Endometrial transcriptome testing can improve the understanding of the endometrial preparation program to guide therapies for specific populations. By using sophisticated genome and molecular biological mechanisms, the understanding of endometrial function can be improved.

## Conclusions

A GnRH agonist combined with HRT did not improve the clinical outcomes of a frozen embryo cycle in older patients. Given that the difference in live births was small, larger studies are needed to determine whether the two methods are equivalent. In addition, many other factors must be considered when determining the optimal endometrial preparation route for an individual. For example, the number of visits, medication, and outpatient expenses, and patient’s expected schedule are important factors in the decision-making process. Clinicians should also discuss with patients the delays that may result from cancelling cycles. These factors should be considered before determining the best choice for increasing the access to treatment for elderly patients. In this population, prospective studies of larger sample sizes are needed.

## Data Availability

Not applicable.

## Abbreviations

FET: Frozen embryo transfer
ART: Assisted reproduction technology
SET: Single embryo transfer
HRT: Hormone replacement treatment
GnRHa-HRT: GnRH antagonist down-regulation combined hormone replacement treatment
RIF: Repeated implantation failure
GnRHa: Gonadotropin-releasing hormone agonist
LH: Luteinizing hormone
TVUS: Transvaginal ultrasound
